# Identification and functional analysis of a bacteriocin, pyocin S6, with ribonuclease activity from a *Pseudomonas aeruginosa* cystic fibrosis clinical isolate

**DOI:** 10.1002/mbo3.339

**Published:** 2016-02-09

**Authors:** Jozef Dingemans, Maarten G. K. Ghequire, Michael Craggs, René De Mot, Pierre Cornelis

**Affiliations:** ^1^ Department of Bioengineering SciencesResearch group Microbiology and VIB Department of Structural BiologyVrije Universiteit BrusselPleinlaan 2Brussels1050Belgium; ^2^ Department Microbial and Molecular SystemsCentre of Microbial and Plant GeneticsKU Leuven Kasteelpark Arenberg 20 ‐ bus 2460HeverleeB‐3001Belgium; ^3^ Present address: AusDiagnostics Pty Ltd205 Victoria StreetBeaconsfieldNSW 2015Australia

**Keywords:** 16S ribonuclease, cystic fibrosis, iron, *Pseudomonas aeruginosa*, pyocins.

## Abstract

S‐type pyocins are bacteriocins produced by *Pseudomonas aeruginosa* isolates to antagonize or kill other strains of the same species. They have a modular organization comprising a receptor‐binding domain recognizing a surface constituent of the target bacterium, a domain for translocation through the periplasm, and a killing or toxic domain with DNase, tRNase, or pore‐forming activity. Pyocins S2, S3, S4, and S5 recognize TonB‐dependent ferri‐siderophore receptors in the outer membrane. We here describe a new nuclease bacteriocin, pyocin S6, encoded in the genome of a *P. aeruginosa* cystic fibrosis (CF) clinical isolate, CF_PA39. Similarly to pyocins S1 and S2, the S6 toxin–immunity gene tandem was recruited to the genomic region encoding exotoxin A. The pyocin S6 receptor‐binding and translocation domains are identical to those of pyocin S1, whereas the killing domain is similar to the 16S ribonuclease domain of *Escherichia coli* colicin E3. The cytotoxic activity was abolished in pyocin S6 forms with a mutation in the colicin E3‐equivalent catalytic motif. The CF_PA39 S6 immunity gene displays a higher expression level than the gene encoding the killing protein, the latter being only detected when bacteria are grown under iron‐limiting conditions. In the S1‐pyocinogenic strain *P. aeruginosa* ATCC 25324 and pyocin S2 producer *P. aeruginosa* PAO1, a remnant of the pyocin S6 killing domain and an intact S6‐type immunity gene are located downstream of their respective pyocin operons. Strain PAO1 is insensitive for pyocin S6, and its S6‐type immunity gene provides protection against pyocin S6 activity. Purified pyocin S6 inhibits one‐fifth of 110 *P. aeruginosa* CF clinical isolates tested, showing clearer inhibition zones when the target cells are grown under iron limitation. In this panel, about half of the CF clinical isolates were found to host the S6 genes. The pyocin S6 locus is also present in the genome of some non‐CF clinical isolates.

## Introduction

*Pseudomonas aeruginosa* is a ubiquitous gram‐negative *γ*‐proteobacterium which has been isolated from various sources, ranging from environmental samples (water, rhizosphere) to humans (Pirnay et al. 2009). In the human host, *P. aeruginosa* can cause disease in immunocompromised as well as cystic fibrosis (CF) patients (Kerr and Snelling 2009; Lyczak et al. 2002). Although the environments from which *P. aeruginosa* strains can be retrieved are diverse, most of them, if not all, share the feature of harboring entire microbial communities (Kent and Triplett 2002; McGuigan and Callaghan 2015; Renwick et al. 2014). These communities are shaped by many symbiotic as well as antagonistic interactions. In order to compete with phylogenetically distant species, *P. aeruginosa* can produce several antagonistic molecules such as phenazines, pyoluteorin, and staphylolysin (LasA protease) among others (Gross and Loper 2009; Kessler et al. 1993). On the other hand, a particular *P. aeruginosa* strain also encounters other pseudomonads within the same niche. To compete with these closely related organisms, *P. aeruginosa* is able to produce bacteriocins. These narrow‐spectrum antibacterial proteins differ from traditional antibiotics, derived from secondary metabolism, in that they generally only affect members of related genera or even the same species. These toxins are generally produced under conditions of stress, such as nutrient depletion or overcrowding (Riley and Gordon 1999; Riley and Wertz 2002a,b).

Bacteriocins produced by *P. aeruginosa* are called pyocins and several types of these bactericidal molecules have been described (Ghequire and De Mot 2014; Michel‐Briand and Baysse 2002). R‐ and F‐type pyocins are particles evolutionary related to bacteriophage tails, hence representing bactericidal tailocins (Ghequire and De Mot 2015; Ghequire et al. 2015). R‐type pyocins are contractile but nonflexible, while the F‐type pyocins are flexible but noncontractile. Another large group of pyocins are the protease‐sensitive “soluble” (S‐type) pyocins. The S pyocins show a modular structure consisting of three domains: a receptor‐binding domain (generally amino‐terminal), a translocation domain, and a killing domain (carboxy‐terminal) (Michel‐Briand and Baysse 2002). Although recently several S‐type pyocins encoding genes have been identified by screening draft and whole‐genome sequences of *P. aeruginosa* strains (Ghequire and De Mot 2014), so far, only six different S‐type pyocins have been functionally characterized, having either DNase (S1, S2, S3, AP41) (Duport et al. 1995; Sano and Kageyama 1993; Sano et al. 1993; Seo and Galloway 1990), tRNAse (S4) (Elfarash et al. 2012) or pore‐forming activity (S5) (Ling et al. 2010). A majority of S‐type pyocins have been found to enter the bacterial cell via binding to a TonB‐dependent outer membrane receptor involved in ferri‐siderophore uptake. More specifically, pyocins S2 and S4 use the ferripyoverdine receptor FpvAI (Denayer et al. 2007; Elfarash et al. 2012), pyocin S3 targets FpvAII (Baysse et al. 1999), while pyocin S5 recognizes the FptA ferripyochelin receptor (Elfarash et al. 2014). Two other pyocin families, comparable in size to the S pyocins, are M‐type and L‐type pyocins. M‐type pyocins host a module that cleaves lipid II peptidoglycan precursors (Barreteau et al. 2009). L‐type pyocins host two monocot mannose‐binding lectin domains, one of which is involved in binding the pseudomonad common polyantigen, and kill target cells via an unknown mechanism (Ghequire et al. 2013, 2014; McCaughey et al. 2014).

Pyocins of the R‐, F‐, and S‐types are produced when the cells are exposed to stress conditions, such as mitomycin treatment (Michel‐Briand and Baysse 2002). The stress‐induced SOS response activates the protease activity of RecA, resulting in the inactivation of the PrtR negative regulator of PrtN, the activator of pyocin genes expression (Matsui et al. 1993; Michel‐Briand and Baysse 2002). Pyocin S5 represents an exception in the sense that its receptor‐binding domain is centrally located and that it is not regulated by the PrtR–PrtN cascade (Ling et al. 2010; Elfarash et al. 2014).

In this study, we identified a novel S‐type pyocin in a CF epidemic *P. aeruginosa* strain, CF_PA39, isolated from a Belgian cystic fibrosis patient (Dingemans et al. 2014). Pyocin S6 shares receptor‐binding and translocation domains with the DNase pyocin S1, but has an rRNase cytotoxic domain similar to the *E. coli* bacteriocin colicin E3. The pyocin S6 toxin gene and cognate immunity gene are located adjacent to the conserved virulence gene *toxA*, in a genomic region where other S‐type pyocins (S1, S2) have been integrated. The pyocin S6 locus is not only found in CF isolates, but is also present in few strains associated with other types of human infections.

## Materials and Methods

### Strains and plasmids

Bacterial strains and plasmids used in this study are described in Table 1. Bacteria were grown at 37°C in Lysogenic broth (LB) medium (Life Technologies) or in iron‐poor Casamino Acids (CAA) medium (Difco Laboratories). Liquid cultures were shaken in a New Brunswick Innova 4000 shaker at 200 rpm.

**Table 1:** Strains and vectors used in this study

Strains or plasmids	Features	References
*Pseudomonas aeruginosa*		
** PAO1**	Wild‐type *P. aeruginosa*	(Stover et al. 2000)
CF_PA1 to CF_PA125^a^	CF clinical *P. aeruginosa* strains	(Dingemans et al. 2014)
*Escherichia coli*		
DH5*α*	*F– Φ80lacZΔM15 Δ(lacZYA‐argF) U169 recA1 endA1 hsdR17 (rK–, mK+) phoA supE44 λ– thi‐1 gyrA96 relA1*	(Hanahan 1985)
BL21(DE3)pLysS	*E. coli* B F^–^ *dcm ompT hsdS*(r_B_^–^ m_B_^–^) *gal* λ(DE3) [pLysS Cam^r^]	(Weiner et al. 1994)
Plasmids		
pET15b	Expression vector, *N*‐terminal his‐tag, Ap^r^	(Studier et al. 1990)
pJB3Tc20	Broad‐host‐range shuttle vector, Ap^r^ and Tc^r^	(Blatny et al. 1997)

a With the exception of CF_PA8, CF_PA18, CF_PA21, CF_PA25, CF_PA29 to CF_PA33, CF_PA52 and CF_PA95 to CF_PA99. Cam^r^ and Ap^r^ indicate resistance to chloramphenicol and ampicillin, respectively

### Structure modeling of the killing domain of pyocin S6

The I‐TASSER server (http://zhanglab.ccmb.med.umich.edu/I-TASSER/) (Yang et al. 2015) was used to acquire a predictive secondary structure of the rRNase domain of pyocin S6. Structure analysis and visualization were performed with PyMOL (www.pymol.org).

### RNA isolation and semiquantitative reverse transcriptase (RT)‐PCR

Using the RNeasy Mini kit (Qiagen), total RNA was isolated from the clinical *P. aeruginosa* strains CF_PA17 and CF_PA39 after 24 h of growth in CAA medium (iron‐limited growth condition) or LB medium (iron‐sufficient growth condition). Prior to reverse transcription, the RNA was treated with Turbo DNase (Ambion) via two 30‐min incubation steps in the presence of 1 *μ*L of (2 U/*μ*L) Turbo DNase. The purity and concentration of the RNA were determined by gel electrophoresis using *oprI* primers (Table S1) (De Vos et al. 1997) and spectrophotometry (NanoDrop, Thermo Scientific). Next, cDNA was synthesized starting from one microgram of Turbo DNase‐treated (Ambion) total RNA using the iScript cDNA synthesis kit (Biorad). A negative control (no reverse transcriptase added) was included for each sample. RT‐PCR was performed on the cDNA templates using primers S6_Fw and S6_Rv, and S6I_Fw and S6I_Rv (Table S1). The PCR conditions included an initial denaturation of 5 min at 95°C, followed by the first of 35 cycles: 45 sec denaturation at 95°C, 45 sec annealing at 55°C, and 2 min extension at 72°C, followed by a final extension at 72°C for 10 min. The PCR products were loaded onto an ethidium bromide‐stained 2% agarose gel using TBE running buffer and visualized under UV light. A Smart Ladder MW‐1700‐10B molecular weight marker (Eurogentec) was run to confirm the expected molecular weight of the amplification product.

### Cloning of the pyocin S6 and S6 immunity genes

The pyocin S6 and adjacent immunity gene (1946 bp) were PCR‐amplified from CF_PA39 genomic DNA using a KAPA HiFi PCR Kit. Primers are listed in Table S1. Amplified fragments were ligated into pET15b (+) (Merck, Germany) after *Nde*I/*Bam*HI double digestion, and introduced in DH5*α* competent cells. A colony PCR using the same primers was performed as selection step for colonies positive for the gene of interest. Plasmids from these positive colonies were purified using the PureYield Plasmid Miniprep system (Promega) and sequenced at the VIB Genetic Service Facility (Wilrijk, Belgium) using two sets of primers (external 715_S6_Fw and 716_S6_Rv, and internal 715_S6_Rv and 716_S6_Fw; Table S1), ensuring complete coverage of the sequence. Pyocin S6 genes, mutated in critical residues in the cytotoxic domain (D533A and E540A), were constructed via splicing by overlap extension (primers in Table S1) and introduced in pET15b (+).

Putative S6 immunity genes of *P. aeruginosa* strains CF_PA39 (350 bp) and PAO1 (370 bp) were PCR‐amplified (primers in Table S1), *Sph*I/*Bam*HI double digested, ligated in shuttle vector pJB3Tc20, and introduced in DH5*α*. Sequence‐verified plasmids were electroporated to indicator strain CF_PA109.

### Overexpression and purification of the S6 protein and S6 mutants

Firstly, the plasmid, of which the sequence was confirmed to contain the genes encoding pyocin S6 and the immunity protein, was introduced into BL21(DE3)pLysS‐competent cells using a heat‐shock protocol. For overexpression, the bacteria with the recombinant plasmids were induced for expression of the cloned gene by growing them overnight at 28°C in the presence of 1 mmol/L IPTG after the OD_600_ reached 0.7. The harvested cells were resuspended in TGE buffer (50 mmol/L Tris‐HCl (pH 7.5), 10% glycerol, 1 mmol/L EDTA, and 10 mmol/L imidazole) and disrupted by French press treatment. The lysate was centrifuged at 10,000*g* for 15 min, and the clear supernatant was loaded onto a His‐Trap FF column (Amersham Biosciences, GE Healthcare) integrated by AKTA TM FPLC system (Amersham Biosciences, GE Healthcare). The His‐tagged proteins were eluted using elution buffer containing 500 mmol/L imidazole in TGE buffer (pH 7.5). The purity of the His‐tagged proteins was confirmed after 12% SDS polyacrylamide gel electrophoresis (SDS‐PAGE, Invitrogen). The gels were stained with Coomassie Blue and a Kaleidoscope protein ladder (BioRad) was used to confirm band size. The purified proteins were dialyzed against 2 L TGE buffer (pH 7.5). The protein concentration was determined using a NanoDrop 1000 spectrophotometer (Thermo Scientific). The pooled pure proteins were divided into small aliquots and stored at −20°C, which were frozen and thawed individually before each manipulation. A similar expression and purification procedure was followed up for the expression and purification of pyocin S6 mutants (D533A and E540A).

### Pyocin sensitivity assay

In order to verify the sensitivity of different clinical isolates (Table 1) to purified pyocin S6, a pyocin spotting assay was performed. This involved spotting 10 *μ*L of the purified pyocin protein (10 mg/mL) onto CAA and LB plates with a bacterial cell layer containing 5 × 10^6^ cells/mL and incubated at 37°C for 24 h. Strains were classified as sensitive if a growth‐inhibitory zone appeared on and/or around the spotting site, and resistant if no such inhibition zone was apparent.

A minimum inhibitory concentration (MIC) test was done in triplicate by spotting a twofold serial dilution (starting concentration, 17 mg/mL) of pyocin S6 onto a lawn of the sensitive *P. aeruginosa* strain CF_PA109 (5 × 10^6^ cells/mL), which was grown overnight on a CAA plate at 37°C for 24 h.

### Screening of clinical strains for the presence of pyocin S6

Chromosomal DNA from our collection of 110 *P. aeruginosa* CF strains (Dingemans et al. 2014) was prepared using the DNeasy Blood and Tissue Kit (Qiagen) and screened for the gene encoding the S6 pyocin using primers S6_Fw and S6_Rv (Table S1). The PCR conditions included an initial denaturation of 5 min at 95°C, followed by the first of 30 cycles: 45 sec denaturation at 95°C, 45 sec annealing at 60°C, and 1 min extension at 72°C, followed by a final extension at 72°C for 10 min.

## Results

### Nucleotide and amino acid sequences of pyocin S6

The genome sequence of *P. aeruginosa* CF_PA39, an isolate belonging to a Belgian epidemic *P. aeruginosa* CF clone (Dingemans et al. 2014), was screened for the presence of existing S‐type pyocin genes. Strikingly, although a coding region corresponding to the receptor‐binding (RBD) and translocation domains (TD) of pyocin S1 (Table 2) was detected in the genome of *P. aeruginosa* CF_PA39, the ORF sequence diverged at the encoded carboxy‐terminal domain, sharing 49.4% amino acid identity and 69% similarity with the rRNase killing domain (KD; Pfam PF09000) of colicin E3 (Table 2). Furthermore, a second ORF overlapping with the putative novel pyocin gene was identified. Considering its genetic organization (ATG start codon overlapping with TGA stop codon of upstream gene) and small size (<100 amino acids), this ORF likely encodes an associated immunity protein, albeit showing only borderline sequence similarity to the colicin E3 immunity protein (85 residues; 18.5 % identity, 37% similarity).

**Table 2:** Comparison of pyocin S6 with other pyocins and colicin E3

Domain/protein	Length (AA)	Pyocin S1 (618 AA)	Pyocin S2 (689 AA)	Pyocin S7 (642 AA)	Colicin E3 (551 AA)
Amino‐terminal	343	99.7	34.7	34.7	NA
Pyocin_S (PF06958)	146	95.8	95.8	99.3	NA
rRNase (PF09000)	82	NA	NA	100	49.4
Immunity^a^	77	NA	NA	100	18.5

The amino acid identity (%) between toxin domains and between cognate immunity proteins is shown. The Pfam domains present in pyocin S6 toxin (571 AA) are specified. NA, not applicable (toxin–immunity pairs of a different type).

a Identity to rRNase immunity orphans (77 AA) in other strains: ATCC 25324 and DK2 (85.7%); PAO1 (84.4%).

**Figure 1:**
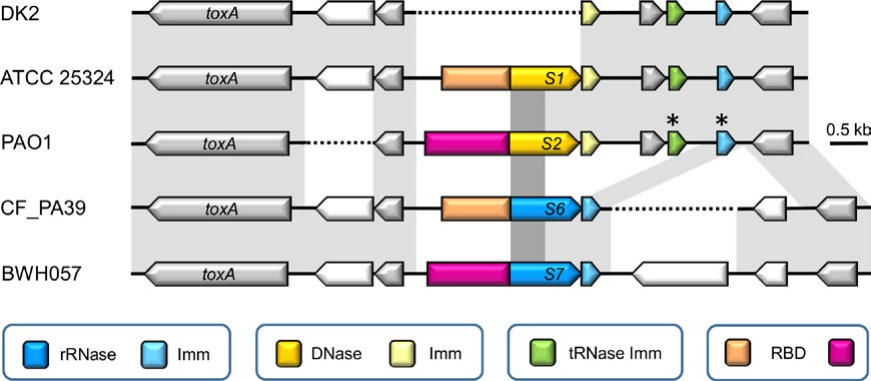
Genetic organization of the pyocin S6 locus in the genome of *P. aeruginosa* CF_PA39. Comparison between the pyocin S6 locus in *P. aeruginosa* CF_PA39 with the loci encoding pyocin S1 (*P. aeruginosa* ATCC 25324), pyocin S2 (*P. aeruginosa* PAO1), and putative pyocin S7 (*P. aeruginosa* BWH057). The equivalent immunity‐encoding genomic region of *P. aeruginosa* DK2 lacking a pyocin toxin gene is included. Synteny is visualized by sequence conservation (light gray shading) for the genes (represented by arrows) located between the orthologs of *toxA* (*PA1148*) and *PAA1153* of *P. aeruginosa* PAO1 (nonannotated ORFs marked with an asterisk). The color code specifies the type of encoded nuclease and cognate or orphan immunity protein (Imm). Conserved receptor‐binding domains (RBD) are highlighted in the same color. Darker gray shading delineates the region encoding the translocation domain, conserved across the four pyocins. Dotted lines indicates the lack of an equivalent sequence. The same pyocin S6 gene context is found in *P. aeruginosa* strains AZPAE14840 and AZPAE14899. In several other S6 pyocinogenic strains the unknown gene upstream of CF_PA39 *toxA*, absent from strain PAO1, is equally lacking (AZPAE14862, AZPAE14931, AZPAE14934, AZPAE14937, AZPAE14951, AZPAE14976, 130_PAER, PA45) (Table S2). The *P. aeruginosa* PA45 locus further differs by the absence of the unknown gene downstream of the CF_PA39 *PA1153* homologue.

Remarkably, the position of the ORFs encoding the putative novel S‐type pyocin (designated S6) and its immunity gene corresponds to the equivalent loci encoding pyocin S1 or pyocin S2 (Fig. 1). In this region between the well‐conserved genes *toxA* and *PA1153* (PAO1 annotation), yet another S‐type pyocin gene pair is found, predicted to encode putative pyocin S7 (Ghequire and De Mot 2014; Fig. 1). The latter apparently combines the RBD of pyocin S2 with the TD and KD of pyocin S6 (Table 2). Downstream of its pyocin S2 locus, *P. aeruginosa* PAO1 harbors a S6‐type immunity gene (previously described as colicin E3 immunity; Denayer et al. 2007), preceded by a 3’‐remnant of the pyocin S6 toxin gene (250 nucleotides), suggesting that the latter was deleted while the putative immunity gene remained intact. A second orphan gene encoding an immunity protein, carrying domain PF09204 and typically associated with a tRNase such as colicin D, is located downstream of PAO1 *PA1152*. Both orphan immunity genes are also located between the *PA1152*‐*PA1153* homologues in the equivalent genomic region of the S1‐pyocinogenic strain ATCC 25324 and in strain DK2. The latter, however, has only retained the DNase immunity gene without a corresponding nuclease gene.

The nucleotide sequence of the two ORFs in the genome of *P. aeruginosa* CF_PA39 spans a region of 1946 nucleotides (Fig. S1). The first ORF encodes a protein of 571 amino acids (pyocin S6) while the second ORF (putative immunity protein of pyocin S6) specifies a small protein of 77 amino acids. The predicted molecular weights of these proteins are 60,901 and 8586 Da, respectively (Fig. S1). The structural domain organization of pyocin S6 was found to be the same as discovered for pyocin S1, with an amino‐terminal polypeptide of 241 amino acids, corresponding to the RBD, a central TD (245 amino acids) and a carboxy‐terminal KD (85 amino acids). As found by Sano et al. (Sano et al. 1993) for pyocin S1, a regulatory P‐box is present 74 nucleotides upstream of the start codon (Fig. S1). The P‐box is located 60–100 bp upstream of the Shine–Dalgarno sequence of the toxin gene and serves as a binding site for the PrtN protein, which positively regulates pyocin expression (Ghequire and De Mot 2014; Matsui et al. 1993). The sequence is usually composed of four repeats of 10–11 nucleotides and each repeat has a consensus sequence of ATTGnn(n)GTnn(n) (Sano et al. 1993). Similar to other characterized S‐type pyocins, a putative ribosome binding site of the immunity gene was found in the region encoding the C‐terminal portion of the toxin protein, located eight bases upstream of the methionine‐encoding start codon. An inverted repeat of eight nucleotides, separated by eight nucleotides, was found downstream of the second ORF, which could serve as a transcription terminator by forming a stem‐loop structure (Fig. S1).

**Figure 2:**
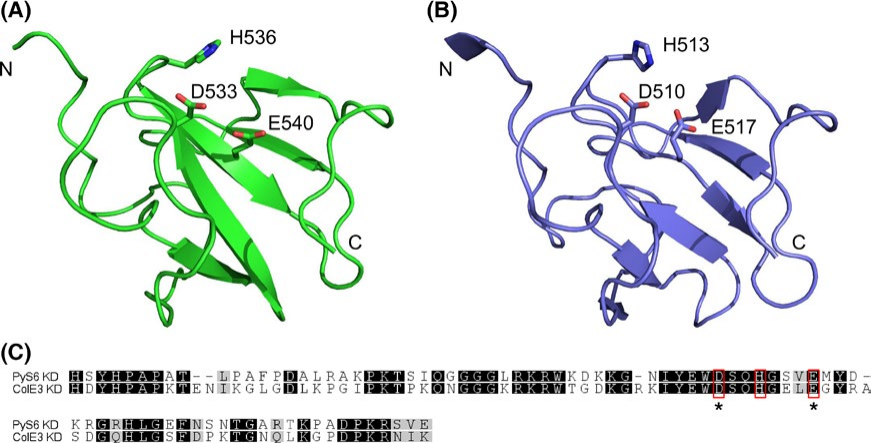
Structural similarity of the killing domains of pyocin S6 and colicin E3. (A). 3D model of the KD of pyocin S6 (B). Cartoon representation of the rRNase KD of colicin E3. Side chains of conserved residues contributing to the activity in colicin E3 (B), and corresponding residues in pyocin S6 (A) are shown as sticks. Amino‐ and carboxy‐termini of the KDs are marked with N and C, respectively. (C). Sequence alignment of the KDs of PyS6 and ColE3. Gray shading reflects the degree of conservation. Residues involved in toxic activity of ColE3, and the corresponding residues in PyS6 are boxed in red. PyS6 residues that were mutated to alanine in this study are marked with an asterisk.

A 3D model of the KD of pyocin S6 was generated using the I‐TASSER server (Fig. 2A). Structural similarity with the KD of colicin E3, for which a crystal structure has been solved, can be observed (Fig. 2B) (Soelaiman et al. 2001). In colicin E3, D510, H513, and E517 are essential residues for bacteriocin activity, whereas a significant reduction in activity was observed when R495, R497, E515, and R545 are mutated (Walker et al. 2004). Interestingly, in pyocin S6, these residues are perfectly conserved (Fig. 2C). Moreover, the orientation of the side chains, expected to contribute to the rRNase activity (D533, H536 and E540, respectively), is highly similar.

### Expression of pyocin S6 and its immunity gene

**Figure 3:**
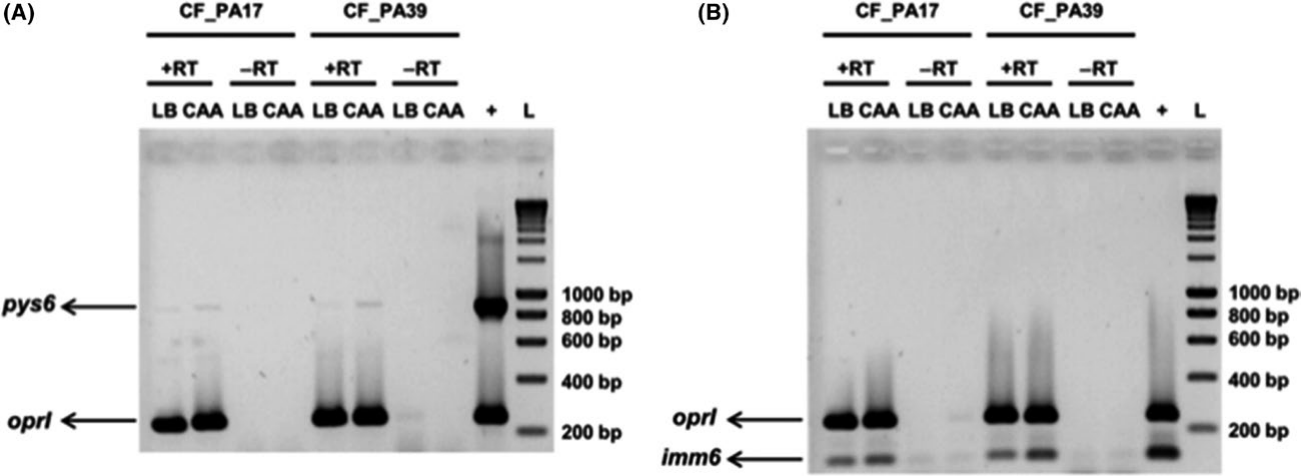
Effect of iron limitation on the expression of *pys6* and *imm6*. Using semiquantitative reverse transcriptase (RT) RT‐PCR, the expression of *pys6* (884 bp) and *oprI* (248 bp) (A) and *imm6* (127 bp) and *oprI* (248 bp) (B) was determined for *P. aeruginosa* CF_PA17 and *P. aeruginosa* CF_PA39 grown in LB or casamino acids (CAA). +RT and −RT indicate that reverse transcriptase was added or not, respectively. Fifty nanograms of genomic DNA isolated from *P. aeruginosa* CF_PA39 was included as a positive control (lanes marked with a “+”). The molecular weight size marker is shown in the lanes marked with an “L”. The lower bands observed in the ‐RT reactions represent primer dimers and are not observed in the +RT reactions. *pys6*, pyocin S6 gene. *imm6,* pyocin S6 immunity gene.

In order to test whether the genes encoding the pyocin S6 and its immunity protein are expressed, we used two different media, LB (iron sufficient) and CAA (iron limited), and a semiquantitative reverse transcription‐polymerase chain reaction (RT‐PCR) was performed (Fig. 3). To determine the relative abundance of expressed genes, primers specific for the housekeeping gene *oprI* were used, using genomic DNA from *P. aeruginosa* CF_PA39 as a positive control. The test was performed on two strains, CF_PA17 and CF_PA39, belonging to the same clone but isolated from different CF patients, both grown overnight in LB or CAA medium.

The obtained results show that signal intensities with regard to the housekeeping *oprI* gene were similar, indicating comparable mRNA levels (Fig. 3). Further, it was found that the expression of the pyocin S6 gene is very low, though slightly increased when the cells were grown in the iron‐limited CAA medium. Strikingly, the expression of the immunity gene was much higher and it could be detected under both growth conditions, suggesting, either an uncoupling between the transcription of the two genes, despite their operon‐like organization or a different stability of the *pyS6* and *imm6* transcripts.

### Cloning, overexpression, and purification of pyocin S6

The DNA sequence encoding both pyocin S6 and its putative immunity protein (1946 bp) was cloned into a pET15b vector. Plasmid pET15b provides an inducible promoter (P*lac*) for cloned genes and allowed expression of the *P. aeruginosa* DNA in *E. coli* BL21 (DE3) pLysS. At an OD_600 nm_ of 0.7 and after 1.5 h of further growth, samples were taken of the *E. coli* culture before and after IPTG induction, respectively, to test for basal expression of the cloned fragment. Subsequently, the samples were analyzed by 12% SDS PAGE. A clear band below 70 kDa (with 63 kDa as expected size of the His‐tagged pyocin S6 protein) was only observed for the induced culture, indicating that the cloned fragment was expressed under control of P*lac* and not under its native promoter. The purified protein appeared as a single band after 12% SDS PAGE (Fig. S2A). The small immunity protein could only be detected when the gel was overloaded (data not shown).

### Pyocin S6 activity

**Figure 4:**
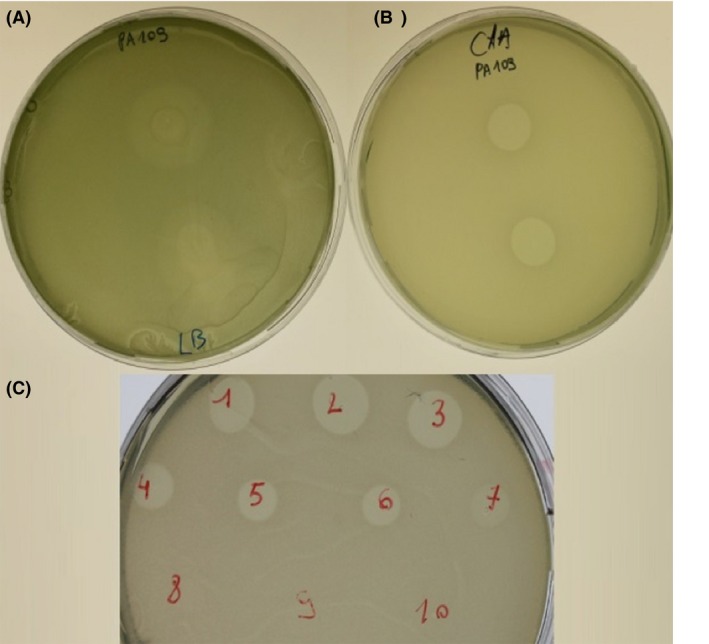
Determination of pyocin S6 activity and MIC (minimum inhibitory concentration). Of the 110 tested strains, 22 were found to be sensitive to the purified protein. Clear zones of inhibition only appeared when grown on casamino acids rather than on LB medium, as demonstrated here for the strain PA109 in figures B and A, respectively. (C) The MIC was determined for CF_PA109 by a twofold serial dilution; number 1 represents the undiluted purified fraction (17 mg mL^−1^). As dilutions increase, the killing zones become smaller and then become gradually more opaque due to incomplete inhibition.

To verify the activity of the purified protein, a sensitivity assay was performed where pyocin S6 was spotted on bacterial lawns of 110 *P. aeruginosa* CF isolates with known ferri‐pyoverdine receptor types (Elfarash et al. 2014), grown on both LB and CAA. Pyocin S6 was found to be active against a total of 22 strains (20%) (Table 3), confirmed by either clear punched‐out zones of no growth or thinning of growth around the spotting area. However, susceptibility was more pronounced under iron limitation, as growth‐inhibitory effects were only clearly visible for strains grown on CAA plates (Fig 4A and B). As the ferri‐pyoverdine receptor types were previously determined for the 110 strains by multiplex PCR (Dingemans et al. 2014), we tried to establish a relationship between the killing activity of pyocin S6 and the presence of a specific ferri‐pyoverdine receptor. However, the results presented in Table 3 do not show any clear correlation, suggesting pyocin S6 does not target a specific ferri‐pyoverdine receptor type.

**Table 3:** Phenotypes of sensitivity or resistance to pyocin S6 of 110 *P. aeruginosa* CF isolates previously checked for the type of FpvA receptor gene by multiplex PCR

	FpvAI	FpvAI + FpvB	FpvAIIb	FpvAIIb + FpvB	FpvAIII	FpvAIII + FpvB
S6 Resistant	0	23	20	16	0	29
S6 Sensitive	2	11	0	7	1	1

To exclude the possibility that the purified pyocin activity could be contaminated by bacteriophages and to establish a MIC, a twofold serial dilution (starting concentration, 17 mg mL^−1^) of pyocin S6 was titrated against the sensitive indicator strain CF_PA109 (Fig. 4C). If any inhibitory activity is due to the presence of bacteriophage, the more diluted fractions will produce individual plaques of phage lysis. If inhibition zones appear without plaque formation, it is assumed that only pyocin activity is present (Osman 1965). No bacteriophage activity was found and the MIC was determined to be 260 *μ*g mL^−1^.

Furthermore, based on the predicted 3D model (Fig. 2), we constructed two mutants in the conserved residues expected to be critical for the rRNase activity in the KD domain of pyocin S6. D533 and E540 in pyocin S6, corresponding to D510 and E517 in colicin E3 (Fig. 2C), respectively, were mutated to alanine. The mutant pyocins were expressed in BL21(DE3)pLysS and purified (Fig. S2A), and spotted onto CF_PA39. As expected, pyocin activity was lost (Fig. S2B).

### Pyocin S6‐downstream gene provides immunity

The putative pyocin S6 immunity gene, accompanied by its cognate ribosome‐binding site, was cloned in shuttle vector pJB3Tc20, and introduced in pyocin S6‐susceptible strain CF_PA109 via electroporation. After spotting pyocin S6 onto a lawn of cells carrying this plasmid, no halo could be observed, whereas CF_PA109 carrying pJB3Tc20 empty vector remained susceptible. This confirms that the pyocin S6‐downstream gene indeed acts as a cognate immunity gene (Fig. S2C and D).

Next, the putative pyocin S6 immunity gene of strain PAO1 was cloned in shuttle vector pJB3Tc20. The encoded PAO1 protein shares 84.4% amino acid identity with the pyocin S6 immunity protein from CF_PA39 (65 identical sites on 77 residues). The upstream DNA region in strain PAO1 is almost identical to CF_PA39, only differing by one nucleotide. The resulting plasmid was electroporated to CF_PA109 and equally tested for pyocin S6 susceptibility. No inhibitory activity could be observed (Fig. S2E), indicating that this rRNase immunity protein provides protection against pyocin S6 as well. Strain PAO1 in insensitive to pyocin S6 activity (data not shown), which is in support of the demonstrated S6 immunity activity.

### Screening of clinical strains for the presence of pyocin S6

PCR analysis was performed to determine the frequency of occurrence of the pyocin S6 gene in the clinical isolates investigated in this study. Fifty‐six of the 110 (51%) strains from the UZ Brussel CF clinic proved to be positive for the gene, explaining in part the relatively low percentage (20%) of susceptible strains. The pyocin S6‐positive cells should be immune due to the constitutive expression of the immunity gene (Fig. 3), providing protection against the exogenous killing protein.

## Discussion

In a recent review, Ghequire and De Mot (2014) presented the result of a screening of different *P. aeruginosa* genomes for the presence of tailocins (R‐ and F‐pyocins) and S‐type pyocins, revealing the presence of uncharacterized S‐type pyocin genes. In this study, we present the functional characterization of such a bacteriocin, pyocin S6, the gene of which was detected in the genome of a CF epidemic strain of *P. aeruginosa*. A comparison with the genomic organization of the pyocin S2 locus in *P. aeruginosa* PAO1 and the pyocin S1 locus of *P. aeruginosa* ATCC 25324 suggests that the pyocin S6 gene has been present in the genome of these strains but got later largely deleted, leaving only the immunity gene intact. In addition to this rRNase‐type of immunity gene, a putative tRNase immunity gene is present downstream of the pyocin S1 and S2 loci. Such orphan immunity genes could confer a competitive advantage to the bacterium as they may enable to neutralize a broader spectrum of invading pyocins as was shown here for the orphan rRNase immunity gene of strain PAO1. Possibly, the pyocin S2 gene was later acquired by horizontal gene transfer. In line with this, an ORF remnant specifying part of a Tn*3* transposon family protein was detected in the PAO1 pyocin S2 locus (Denayer et al. 2007).

The similarities in tertiary structures, together with the 53% identity of the KDs and the conservation of catalytic residues, suggest that in target cells, pyocin S6 uses a killing action comparable to colicin E3 by inactivation of the host's protein biosynthetic machinery through cleavage of a single ribonucleotide bond at the ribosomal A‐site of 16S rRNA. This mode of action likely results in a bacteriostatic effect rather than a bactericidal activity as for example mediated by the DNAse killing domain of S2.

The fact that pyocin S6 shares similarity with colicins is not so surprising, since the same has been found for pyocins S1, S2, and AP41, where the DNase domains and the immunity proteins of these pyocins were found to be homologous to the corresponding regions of E2 group colicins; E2, E7, E8, and E9 (Sano et al. 1993). This demonstrates that, over the course of evolution, pyocins have been subjected to forms of recombination and domain shuffling events with colicins and possibly bacteriocins of other bacteria. A conceivable explanation for this phenomenon can be found in the clear structural organization that both bacteriocins have in common, even though the putative domains of pyocin S6 (RBD, TD, and KD) are differently arranged compared to those in colicin E3, in which the order is TD‐RBD‐KD from amino‐ to carboxy‐terminal end. Similarly to other S‐type pyocins, pyocin S6 could possibly targets sensitive cells by binding to an iron‐regulated outer membrane receptor. Attempts to identify the receptor recognized by the RBD using the same strategy as for the identification of the FptA receptor for pyocin S5 (Elfarash et al. 2014) did not succeed. In the case of pyocin S5, a combined library of transposon mutants of an S5‐sensitive strain was subjected to the action of S5 and resistant colonies were found growing in the pyocin inhibition zone. This failure is probably due to the bacteriostatic activity of pyocin S6, which allows regrowth of cells in the inhibition zone after a longer incubation period, hence masking the presence of true resistant clones after transposon mutagenesis.

Interestingly, the expression levels of the immunity gene are considerably higher than those of the pyocin S6 gene under both growth conditions used in this study. This seems contradictory to the operon‐like genomic organization of the genes. However, it has been shown that the expression of genes in an operon can be discoordinately regulated by means of small noncoding RNAs (sRNAs) (Balasubramanian and Vanderpool 2013). In the case of pyocin S6, such a discoordinate regulatory mechanism might protect the bacterial cell from spending energy in the production of the pyocin molecule, while under iron‐limiting conditions, when the target receptors are abundantly expressed, a different regulatory mechanism may stabilize the pyocin transcript. On the other hand, high basal expression of the immunity gene protects the pyocin‐producing cell against invading pyocins, and in addition, ensures that it can rapidly respond to changing conditions. In a recent study, Gomez‐Lozano et al. (2014) identified by RNA‐seq analysis, 232 antisense RNAs in *P. aeruginosa* PAO1, one in the pyocin S5 gene, and three of them internal of the pyocin S4 coding sequence.

In our PCR‐based screening of 110 CF strains of *P. aeruginosa*, we found that 51% of the panel contains the pyocin S6 gene. It should be mentioned that in this collection of *P. aeruginosa* CF strains, there is a bias toward strains belonging to the *P. aeruginosa* CF_PA39 clone (20 out of the 110 tested isolates), explaining the high frequency of isolates harboring the pyocin S6 gene and consequently the lower number of sensitive isolates, as compared to susceptibility frequencies for other pyocins. However, when screening the *Pseudomonas* Genome Database (Winsor et al. 2011) via BlastN, the complete pyocin S6 gene could not be retrieved from any of the available complete *P. aeruginosa* genomes, and was found in only six strains out the 996 complete and draft genomes (Table S1), including CF_PA39, two strains from respiratory infections, one abdominal infection isolate, one blood isolate, one urinary tract infection (UTI) isolate, and one tomato plant isolate.

## Conclusion

This study has provided the first experimental evidence of a locus encoding a novel *P. aeruginosa* pyocin, termed pyocin S6, and its cognate immunity domain in the genome of a cystic fibrosis isolate. The genes for this particular pyocin and its immunity protein are found in only few other clinical isolates, and even one nonclinical isolate, but are prominent in the strains belonging to the same CF clonal complex from the UZ Brussels. Sharing two domains with pyocin S1 (RBD and TD), its toxic activity is quite similar to that of colicin E3, a bacteriocin from *E. coli* that kills through site‐specific cleavage of a single phosphodiester bond of 16S ribosomal RNA in the 30S subunit. In other *Pseudomonas* species, mainly isolated from plant and soil environments, putative bacteriocins with a colicin E3/pyocin S6‐type of carboxy‐terminal domain occur frequently (Ghequire and De Mot, 2014). These molecules illustrate the significance of domain shuffling in molecular evolution.

## Conflict of Interest

None declared.

## Supporting information


**Figure S1.** Detailed overview of the *pys6*/*imm6* locus. The stop codon of the *pys6* gene is underlined once, while the start codon of *imm6* is underlined twice.

**Figure S2.** (A) SDS PAGE electrophoresis of purified pyocin S6 and mutants. Lane 1, Kaleidoscope size marker (kDa); lane 2, pyocin S6; lane 3, pyocin S6 with D533A; lane 4, pyocin S6 with E540A.

**Table S1.** Primers used in this study.
