# Supplementary figures and images for: Identification and functional analysis of a bacteriocin, pyocin S6, with ribonuclease activity from a Pseudomonas aeruginosa cystic fibrosis clinical isolate
Source: Microbiologyopen. 2016 Feb 9;5(3):413–23. doi: 10.1002/mbo3.339 (PMC4905994; doi:10.1002/mbo3.339)

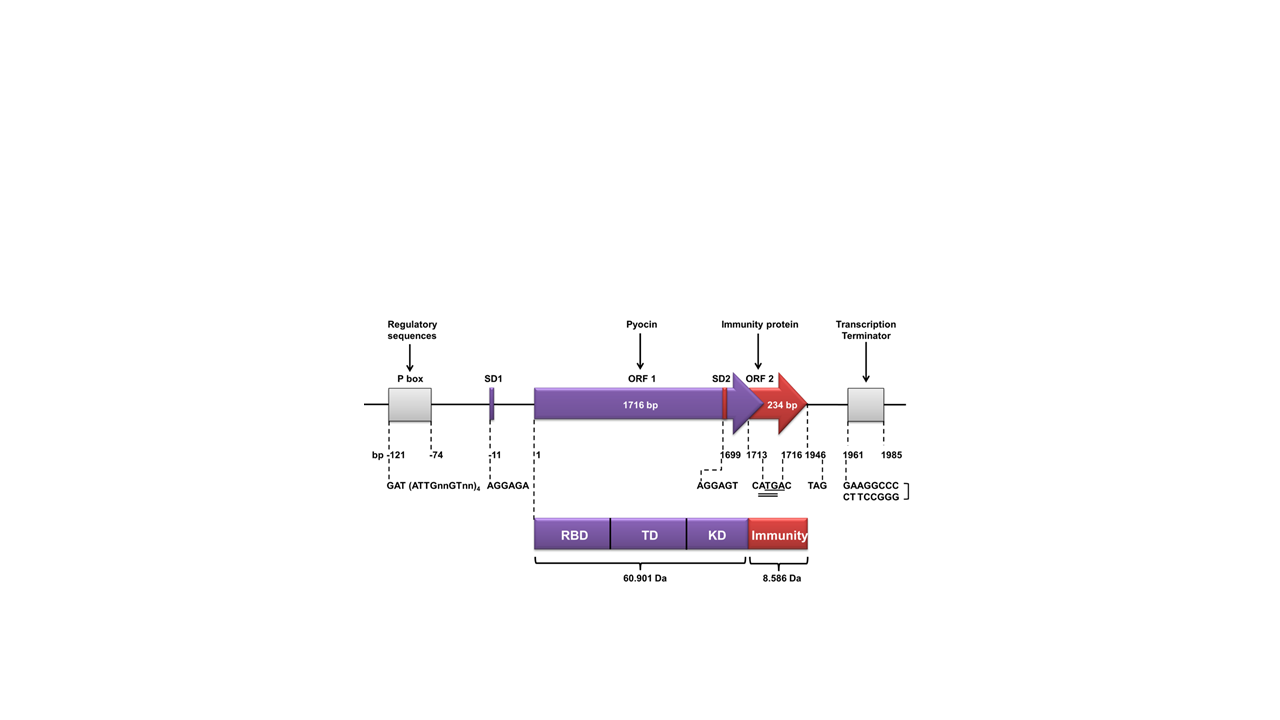

Supplement: Supplementary file 1 — Figure S1. Detailed overview of the pys6/imm6 locus. The stop codon of the pys6 gene is underlined once, while the start codon of imm6 is underlined twice. [file MBO3-5-413-s001.tif]

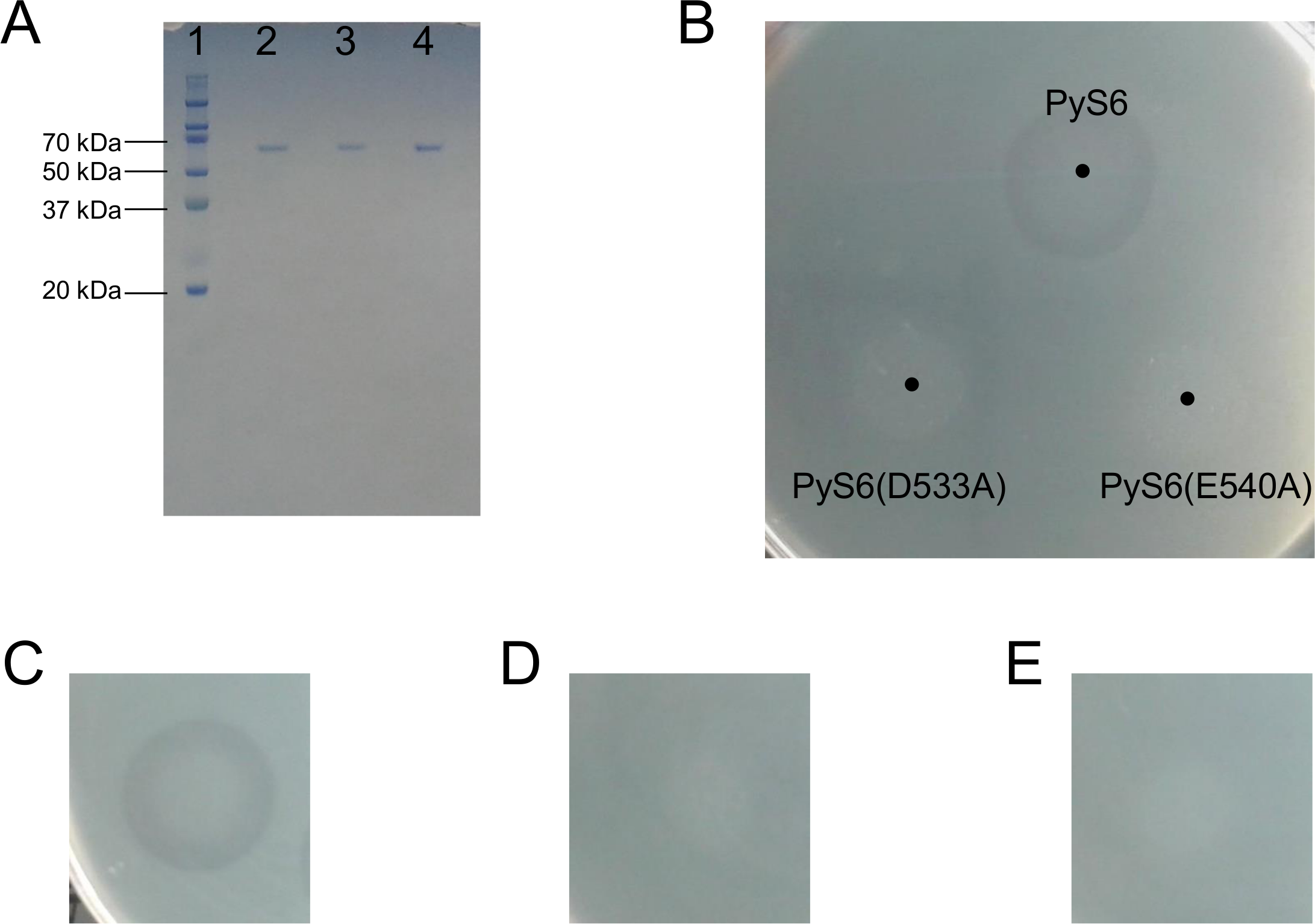

Supplement: Supplementary file 2 — Figure S2. (A) SDS PAGE electrophoresis of purified pyocin S6 and mutants. Lane 1, Kaleidoscope size marker (kDa); lane 2, pyocin S6; lane 3, pyocin S6 with D533A; lane 4, pyocin S6 with E540A. [file MBO3-5-413-s002.tif]
