# Supplementary material for: Identification and functional analysis of a bacteriocin, pyocin S6, with ribonuclease activity from a Pseudomonas aeruginosa cystic fibrosis clinical isolate
Source: Microbiologyopen. 2016 Feb 9;5(3):413–23. doi: 10.1002/mbo3.339 (PMC4905994; doi:10.1002/mbo3.339)
Supplement: Supplementary file 3 — Table S1. Primers used in this study. [file MBO3-5-413-s003.docx]

**Table S1.** List of primers used in this study

| Name | Primer sequence (5`→ 3`) | RE | Product size |
| --- | --- | --- | --- |
| Pyocin S6 primers |  |  |  |
| S6_Fw | GTCTCCAGATCCGCATGAAT | - | 884 bp |
| S6_Rv | CGGAGCAGGATGGTAACTGT |  |  |
| Immunity primers |  |  |  |
| S6I_Fw | CCTAGCATCGGGAAATGATG | - | 127 bp |
| S6I_Rv | TACTCCAATCCAACCGGAAG |  |  |
| Housekeeping gene primers | |  |  |
| oprI_Fw | ATGAACAACGTTCTGAAATTCTCTGCT | - | 248 bp |
| oprI_Rv | CTTGCGGCTGGCTTTTTCCAG |  |  |
| Pyocin S6 cloning primers |  |  |  |
| S6C_Fw | GGAATTCCATATGGCACGACCCATTGCTGACCTTA | *Nde1* | 1946 bp |
| S6C_Rv | CGGGATCCCTAGGCGTAAACCCCAATAAAATAC | *BamH1* |  |
| KD mutation primers |  |  |  |
| S6-D533A_FW | AATATCTATGAATGGGCCTCTCAGCATGGCAGT |  |  |
| S6-D533A_RV | ACTGCCATGCTGAGAGGCCCATTCATAGATATT |  |  |
| S6-E540A_FW | CAGCATGGCAGTGTTGCAATGTATGACAAGAGA |  |  |
| S6-E540A_RV | TCTCTTGTCATACATTGCAACACTGCCATGCTG |  |  |
| S6 immunity cloning primers |  |  |  |
| S6ImmCFPA39_FW | TGGCTAGCATGCCTGATCCAAAAAGGAGTGTAGAACC | *Sph*I | 350 bp |
| S6ImmCFPA39_RV | TGGCTAGGATCCTCCACCAATAGCTGTCCAACT | *Bam*HI |  |
| S6ImmPAO1_FW | TGGCTAGCATGCCTGATCCAAAAAGGAGTGTAGAAGC | *Sph*I | 370 bp |
| S6ImmPAO1_RV | TGGCTAGGATCCCAAGGAAGAGCACTCTATTGCC | *Bam*HI |  |
| Pyocin S6 sequencing primers |  |  |  |
| 715_S6_Fw | TAATACGACTCACTATAGGG | - | 1133 bp |
| 715_S6_Rv | AGGGCGTAACGAACGCTAT |  |  |
| 716_S6_Fw | GGCTTTGCCAGTCTGACCTA | - | 1106 bp |
| 716_S6_Rv | GCTAGTTATTGCTCAGCGG |  |  |
| pJB3Tc20 sequencing primer |  |  |  |
| PGPRB-8461 | GCTCACTCATTAGGCACCC |  |  |

The underlined sequences, added to the primer fragments, indicate the recognition sites of the restriction enzyme (RE) used for the cloning experiments.
